# A FreeSurfer view of the cortical transcriptome generated from the Allen Human Brain Atlas

**DOI:** 10.3389/fnins.2015.00323

**Published:** 2015-09-16

**Authors:** Leon French, Tomáš Paus

**Affiliations:** ^1^Rotman Research Institute, BaycrestToronto, ON, Canada; ^2^Departments of Psychology and Psychiatry, University of TorontoToronto, ON, Canada; ^3^Center for the Developing Brain, Child Mind InstituteNew York, NY, USA

**Keywords:** FreeSurfer, gene expression, cortex, neuroanatomy, atlas, transcriptome

## Introduction

The Allen Human Brain Atlas provides an anatomically comprehensive view of gene expression in the brain (Hawrylycz et al., 2012). The complete transcriptome dataset consists of 58,692 measurements of gene expression in 3702 brain samples obtained from 6 individuals. The resulting product, over 200 million gene expression values, can be overwhelming for neuroscientists seeking to use the data. For example, data for a single gene consist of three interlinked files when downloaded from the Allen Institute website. The local expression values are associated with X, Y, and Z coordinates in the MNI152 atlas (Mazziotta et al., 2001), thus allowing one to map these values to other brain atlases.

To reduce the complexity of the Allen data, we have summarized the data into the Desikan–Killiany cortical atlas built into the FreeSurfer software for automatic labeling of regions of interest (Desikan et al., 2006). FreeSurfer allows one to segment magnetic resonance images (MRIs) into 68 cortical regions and to estimate their cortical thickness, surface area and volume (http://surfer.nmr.mgh.harvard.edu/). These cortical measurements are stable and agree with past histological studies (Fischl and Dale, 2000; Han et al., 2006; Scholtens et al., 2015). FreeSurfer has been used widely, with over 600 reports covering a broad range of topics that include brain development, aging and a variety of brain disorders (PubMed, May 2015). Adding the perspective of gene expression could facilitate interpretation of these reports. For example, we recently found that the magnitude of group differences in cortical thickness between cannabis users and non-users correlates with regional variations in expression levels of cannabis receptor 1 (*CNR1*) (French et al., 2015).

## Methods and materials

Complete microarray gene expression datasets were downloaded from the Allen Institute of Brain science website (http://human.brain-map.org/static/download/, downloaded May 2015). These datasets were obtained from six individuals (five males, one female), with age ranging from 24 to 57 years. Gene expression was assayed by the Allen Institute with custom designed Agilent arrays. Blockface images of tissue slabs were used to map samples of gene expression to anatomical locations. Before dissection, whole brain MR images were obtained for each brain (Hawrylycz et al., 2012). The MR images were registered to the MNI152 space using the FreeSurfer affine registration method for the two *in cranio* brains and a deformable technique used for the four *ex cranio* brains. The above steps were carried out by the Allen Institute. Details of the methods and quality controls used by the Allen Institute are available in the Microarray Survey Technical White paper (http://help.brain-map.org/display/humanbrain/Documentation).

After downloading the raw data (expression values plus the MNI152 X, Y, and Z coordinates for each brain location), we have carried out the following data-reduction steps.

The first stage of data reduction was performed at the probe level: expression values from multiple probes were mean averaged for each gene (using the annotations provided in Probes.csv).

The second stage involved spatial mapping of the samples to the FreeSurfer cortical regions (Desikan et al., 2006). First, we used the Allen Institute anatomical annotations to remove samples located outside the cerebral cortex. Then, we used the MNI152 coordinates (in conjunction with the anatomical annotations) to determine if a specific sample was contained in a FreeSurfer cortical region. In order to map the MNI152 coordinates (Allen Atlas) into the FreeSurfer space, we ran FreeSurfer 5.3 on the MNI152 template to produce sets of MNI152 coordinates (voxels) corresponding to each of the FreeSurfer cortical regions.

A sample was mapped automatically to a FreeSurfer region if its MNI 152 sample coordinates were inside a FreeSurfer cortical region voxel set (or within one voxel). These automatic assignments were reviewed for their accuracy by visual examination of the voxel's location and checking the assigned region names (for example “ctx-lh-middletemporal” matches “middle temporal gyrus, Left, inferior bank of gyrus”). More extensive review was used for the cortical Allen samples not directly inside a FreeSurfer cortical region voxel set (56% of all cortical samples). For this review, we also considered an anatomic annotation of the closest FreeSurfer cortical region; the Allen Reference Atlas annotations were given, however, more weight in these mapping decisions.

**Figure 1:**
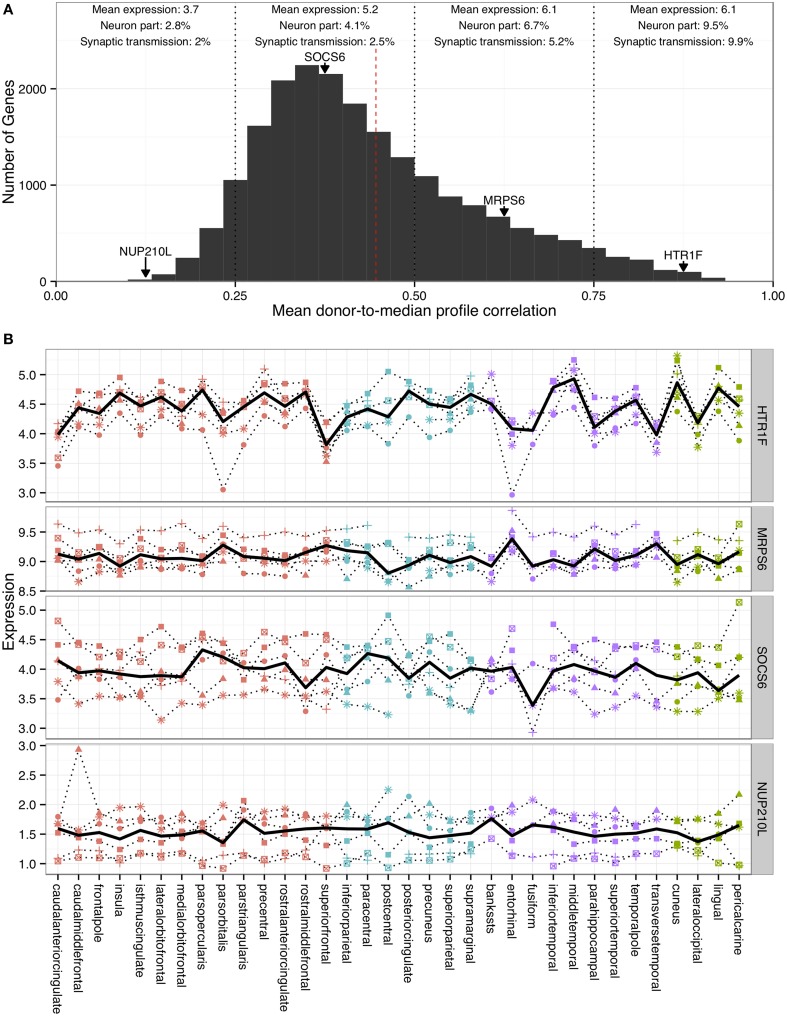
**Characterization of correlations between individual donor profiles and the median profile. (A)** Histogram of donor-to-median profile correlations. The dashed red line marks a correlation of 0.446, which corresponds to one sided *p* < 0.05 (derived from random simulations of donor expression profiles). Mean expression levels and overlaps with the “synaptic transmission” and “neuron part” gene ontology groups are provided for genes in four correlation ranges (marked as dotted vertical lines). Expression profiles for four representative genes that lie at the midpoints of each range are provided in **(B)** and marked in **(A)** with arrows (*HTR1F, MRPS6, SOCS6*, and *NUP210L*). The solid black line represents the median profile and the six donor profiles as dotted lines in **(B)**. Each region is color-coded to indicate its cortical lobe: frontal (red), parietal (blue), temporal (purple) and occipital (green). Each symbol shape represents a specific donor.

The third stage involved averaging values of gene expression across all voxels mapped into a specific FreeSurfer region; for each individual brain, median values were obtained for each gene and each cortical region. These values were then summarized across the individual brains; for the group of six brains, median values were calculated for each gene and region (see Figure 1 for examples). The result is seven gene-expression profiles (six donors and one median profile). These profiles can be represented as a vector of regional expression values for each gene. Mean Spearman correlation between these vectors (or profiles) of the individual brains and the median result is used to provide a measure of variance across the individuals for a specific gene (referred to as consistency).

### Gene ontology enrichment analysis

The ErmineJ software (version 3.0.2) was used for Gene Ontology enrichment analyses (Gillis et al., 2010), genes were ranked according to the consistency measure to generate receiver-operator curves for all gene ontology groups in the molecular process and cellular component ontologies with more than 10 and less than 1000 genes (5148 groups)(Ashburner et al., 2000). The gene ontology annotation version was dated December 2014. More recent annotations for the “neuron part” and “synaptic transmission” groups were downloaded from the AmiGO website for use in Figure 1 (Carbon et al., 2009).

### Visualization in TkSurfer FreeSurfer tool

We provide an R script to convert the gene expression data into 3d images (CreateExpressionLUT.r). This script produces a gene-specific color lookup table (LUT) for the FreeSurfer annotation files ([lr]h.aparc.annot). Instructions for loading the LUT file in TkSurfer are provided at the end of the script.

## Results

Averaging the 58,692 probes to genes results in expression data for 20,737 genes (8394 probes are not annotated to a gene). Of the 3702 brain samples, 1950 were annotated to regions located in the cerebral cortex. Using the MNI152 coordinates, 866 regions (44% of all cortical samples) were contained in a FreeSurfer region (or within one voxel from the region). Further operator-based (CG, LF, TP) annotation expanded this set to 1697 mapped samples. Using this mapping, we have calculated median expression profiles for each of the 20,737 genes. These profiles are vectors of average expression of a gene across the 68 cortical regions (AllenHBA_DK_ExpressionMatrix.tsv). This matrix can be easily loaded into R or a spreadsheet program to provide a summary view for any gene of interest.

Given the limited availability of data for the right cerebral hemisphere (2 of 6 individuals), we focus henceforth on the left hemisphere in which gene expression data are available for all six donors (1269 samples mapped to 34 left-hemisphere cortical regions). The number of donors per region varied between all six donors for 28/34 regions to three donors for 1/34 region. The number of samples per region ranged from 6 (bankssts) to 100 (superiorfrontal) (details in DKRegionStatistics.tsv). For each gene, we correlated the generated expression profiles of the six donors to the median profile (left hemisphere regions only, 34 regions). The average of these donor-to-median profile correlations provides a measure of consistency across the six brains (data in AllenHBA_DK_ExpressionMatrix.tsv). Using this metric, the median profile provides a good approximation across the donors for 39.7% of the assayed genes (Spearman's ρ > 0.446 corresponding to one sided *p* < 0.05, derived from random simulations of donor expression profiles). Figure 1 shows the distribution of this consistency measure and profiles of four representative genes. Across the genome, consistency is correlated with average expression of a gene (Spearman's ρ = 0.29, *p* < 0.001) and variance across the regions for a given gene (Spearman's ρ = 0.48, *p* < 0.001). In agreement, housekeeping genes that are expressed at a constant level across tissues have lower donor-to-median profile correlations (*p* < 0.001) (Eisenberg and Levanon, 2013). Genes with high consistency are enriched Gene Ontology groups associated with “neural” processes and components (ConsistentGOGroups.tsv). Groups with the highest consistencies from the biological process aspect include “synaptic transmission” (GO:0007268, corrected *p* < 0.001, 441 genes) and “neuron part” from the cellular component aspect (GO:0097458, corrected *p* < 0.001, 769 genes). Overlaps with these groups for each quarter of the consistency range are provided in Figure 1A. Groups with the lowest consistencies include “detection of chemical stimulus involved in sensory perception of smell” (GO:0050911) and “intermediate filament cytoskeleton” (GO:0045111) (InconsistentGOGroups.tsv). These groups contain mostly olfactory receptor and keratin genes respectively, which do not appear to be expressed in the cortex (Lonsdale et al., 2013).

**Figure 2:**
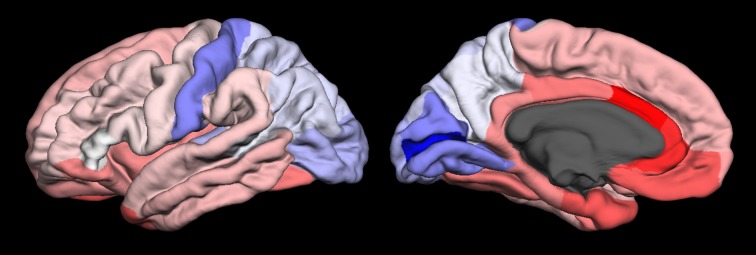
**Lateral (left) and medial (right) views of *CNR1* gene expression data projected onto the FreeSurfer cortical regions in the left hemisphere of the fsaverage brain template**. Expression (log2 intensity) ranges from 5 (blue), 5.92 (white), and red (6.83).

Figure 2 presents a cortical surface map of *CNR1* expression. This example image was created using our R script that creates color lookup tables for the TkSurfer tool (CreateExpressionLUT.r). In the case of *CNR1*, consistency across the six individual brains is high (Spearman's ρ = 0.82, 98 percentile). This figure represents a summarized and filtered view of 329,478 *CNR1* expression measurements provided in the Allen Brain Atlas and processed with the tools described in this report; creating this figure involved downloading two files and executing the R script, all provided in this report. While similar visualizations are provided by the Allen Institute website (http://human.brain-map.org/) and Brain Explorer 2 3D viewer they do not provide a single view that summarizes across donors, probes or FreeSurfer cortical regions (Figure S1). We hope that our technique will help facilitate interpretation of neuroimaging findings in a molecular context.

## Discussion

For over 60% of the assayed genes, donor-to-median profile correlations were not significantly different from those of randomly generated expression profiles. These genes may have more consistent expression profiles in the subcortical brain regions that were assayed in the Allen Institute data. Genes with low consistency are possibly more subject to influences of postmortem interval, agonal state, brain pH and age at death (Preece and Cairns, 2003). We note, however, that the less than a thousand genes were correlated with these factors in meta-analyses of post-mortem cortex (Mistry and Pavlidis, 2010). These variables were found to have little impact on array quality and expression profiles (Trabzuni et al., 2011). Across the genome, inconsistent genes have constant (housekeeping genes) or low expression across the cortex (olfactory receptors and keratins). These properties possibly result in low correlations across the donors due a lower signal to noise ratio. Reassuringly, neural associated genes show consistent spatial patterns across the six brains.

To demonstrate further the utility of the new resource, we discuss two recent studies and suggest new hypotheses that could be tested with the data reported here. In the first study, differences in cortical thickness across the FreeSurfer regions were calculated between healthy controls and patients with bipolar disorder taking (or not) lithium (Giakoumatos et al., 2015). One could hypothesize that the reported spatial patterns of these differences are correlated with the expression patterns of genes that respond to lithium ions. A second study analyzed connectivity matrices obtained from healthy controls and patients with schizophrenia using the FreeSurfer-based parcellation (van den Heuvel et al., 2013). These matrices could be easily merged with the gene-expression data provided here using the same matrix structure; for example, one could then compare the nodes with large group differences in structural or functional connectivity vis-à-vis local (or correlated) gene expression.

## Overview of the data files and their formats

Five data files and one script that accompany this report are available at the figshare repository (French, 2015) (http://figshare.com/articles/A_FreeSurfer_view_of_the_cortical_transcriptome_generated_from_the_Allen_Human_Brain_Atlas/1439749).

AllenHBA_to_DKRegion_Map.xls—Excel file containing the mapping from Allen Brain Atlas samples to the FreeSurfer cortical regions. Each sample is referenced with a unique identifier by combining the donor ID with the x, y and z MNI152 coordinates (“10021_5.1_27.1_28.6” for example).

DKRegionStatistics.tsv—Tab separated file characterizing the FreeSurfer cortical regions. This file lists how many donors contribute to each region, Allen Brain Atlas samples per region and alternative identifiers.

AllenHBA_DK_ExpressionMatrix.tsv—Tab separated file containing correlation to the median values across the donors (left hemisphere only, column named “Average donor correlation to median”) and gene expression values across the 68 FreeSurfer cortical regions (columns). This file can be opened with read.table in R or as a tab separated file in a spreadsheet program.

ConsistentGOGroups.tsv—Gene Ontology enrichment analyses results for the consistency measure.

InconsistentGOGroups.tsv—Gene Ontology enrichment analyses results for the consistency measure (with high ranked groups showing more inconsistency).

CreateExpressionLUT.r—This R script provides the method to load and convert expression values for a specific gene into a color lookup table that can be used to visualize the averaged expression values in FreeSurfer.

### Conflict of interest statement

The authors declare that the research was conducted in the absence of any commercial or financial relationships that could be construed as a potential conflict of interest.

## Supplementary material

Five data files and one script that accompany this report are available at the figshare repository (http://figshare.com/articles/A_FreeSurfer_view_of_the_cortical_transcriptome_generated_from_the_Allen_Human_Brain_Atlas/1439749)

Figure S1 **Lateral view of *CNR1* gene expression data in the Allen Institute's Brain Explorer 2 software**. Only the left hemisphere cortical regions are shown. The CUST_1103_PI417557136 probe was selected because it's most correlated with the average of the 89 CNR1 probes.
