# Supplementary figures and images for: A FreeSurfer view of the cortical transcriptome generated from the Allen Human Brain Atlas
Source: Front Neurosci. 2015 Sep 16;9:323. doi: 10.3389/fnins.2015.00323 (PMC4584957; doi:10.3389/fnins.2015.00323)

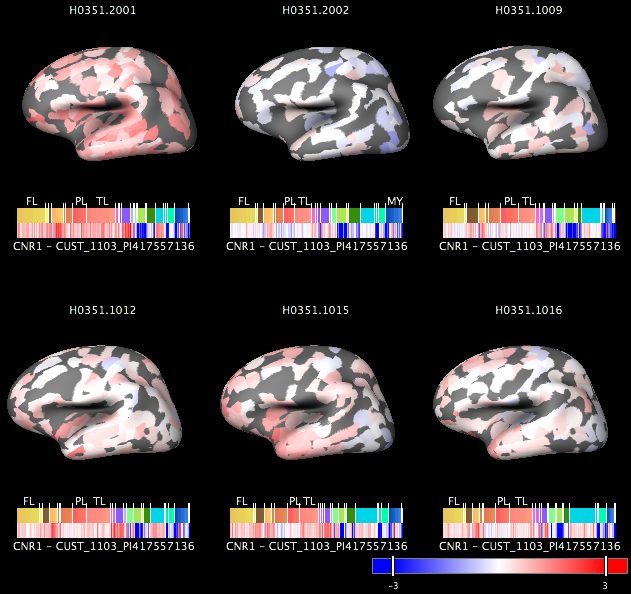

Supplement: Figure S1 — Lateral view of CNR1 gene expression data in the Allen Institute's Brain Explorer 2 software. Only the left hemisphere cortical regions are shown. The CUST_1103_PI417557136 probe was selected because it's most correlated with the average of the 89 CNR1 probes. [file FigureS1.png]
